# Advancing Multi-Touch Sensing: Integrating FTIR and ToF Technologies for Precise and Large-Scale Touch Interfaces

**DOI:** 10.3390/s25175503

**Published:** 2025-09-04

**Authors:** Andrejs Ogurcovs, Ilze Aulika, Sergio Cartiel, Meldra Kemere, Jelena Butikova, Eriks Sledevskis

**Affiliations:** 1 Institute of Solid State Physics, University of Latvia, Kengaraga Street 8, LV-1063 Riga, Latvia; ilze.aulika@cfi.lu.lv (I.A.); meldra.kemere@cfi.lu.lv (M.K.); jelena.butikova@cfi.lu.lv (J.B.); 2 Instituto de Investigación en Ingeniería de Aragón (I3A) Zaragoza, University of Zaragoza, C. Maria de Luna, 1, 50019 Zaragoza, Spain; scartiel@unizar.es; 3 G. Liberts’ Innovative Microscopy Centre, Department of Technology, Institute of Life Sciences and Technology, Daugavpils University, Parades Street 1A, LV-5401 Daugavpils, Latvia; eriks.sledevskis@du.lv

**Keywords:** light detection and ranging, LIDAR, Time-of-Flight, touch detection, tactile sensing, frustrated total internal reflection, FTIR, wave guide, light guide, optical sensing, robotic sensor skin, proximity sensing

## Abstract

Building upon recent advances in tactile sensing platforms such as OptoSkin, this research introduces an enhanced multi-touch sensor design that integrates Frustrated Total Internal Reflection (FTIR) technology with embedded Time-of-Flight (ToF) sensors for superior performance. Utilizing a 2 mm thick poly(methyl methacrylate) (PMMA) acrylic light guide with an area of 200 × 300 mm^2^, the system employs the AMS TMF8828 ToF sensor both as the illumination source and the receiver. The selected PMMA, with a refractive index of 1.49, achieves an optical field of view (FoV) of approximately 32 degrees for the ToF receiver and enables signal propagation with minimal optical loss. Remarkably, a single ToF sensor can cover an active area of 195 cm^2^ with a linear resolution of approximately 1 cm and an angular resolution of up to 3.5 degrees. This configuration demonstrates not only the feasibility of direct FTIR–ToF integration without the need for external cameras or electrode arrays but also highlights the potential for precise, scalable, and cost-effective multi-touch sensing over large surfaces. The proposed system offers robust performance even under direct sunlight conditions, setting a new benchmark for advanced tactile interface development across consumer electronics, industrial control, and robotic skin applications.

## 1. Introduction

Multi-touch sensing has become a foundational technology in modern human–machine interfaces, with applications ranging from smartphones and interactive displays to industrial panels and robotic controls. Among the various sensing modalities—capacitive, resistive, ultrasonic, and optical—Frustrated Total Internal Reflection (FTIR) has emerged as a robust and precise method, notable for its mechanical simplicity, high spatial resolution, and immunity to ambient electromagnetic noise [1]. FTIR systems rely on the disruption of internally reflected light within a transparent waveguide, typically glass or polymer, where user interaction scatters the evanescent field, which can then be captured optically [2,3].

Since its introduction into interactive systems by Han [1], FTIR technology has undergone extensive evolution. Early designs used infrared cameras to capture touch points, enabling low-cost multi-touch surfaces but imposing practical limitations due to system bulk, calibration complexity, and sensitivity to environmental lighting. Later implementations, including FLATIR [3] and custom optical stacks [4], enhanced accuracy and compactness by using discrete photodiodes or LEDs. More recently, innovations in waveguide materials and coupling techniques have allowed for thin, flexible FTIR configurations compatible with curved and wearable displays [5,6].

Despite these advances, traditional FTIR systems still face challenges in scalability, robustness, and integration. Vision-based designs are difficult to miniaturize and require considerable processing power. Additionally, achieving uniform illumination and precise calibration across large or complex surfaces remains difficult. To overcome these limitations, recent research has proposed hybrid architectures combining FTIR principles with embedded optical time-of-flight (ToF) sensing [7,8]. These designs, such as the OptoSkin platform, replace external cameras with LiDAR-based sensors to detect touch-induced light scattering directly within the waveguide—enabling depth-aware, camera-free, and low-power tactile sensing.

In this work, we present a detailed experimental characterization of such an FTIR–ToF hybrid system. Our prototype uses a planar poly(methyl methacrylate) (PMMA) waveguide and TMF8828 direct ToF sensors to isolate and analyze the fundamental signal behaviors in a quasi-ideal configuration. PMMA was selected for its high optical transparency, low scattering coefficient (0.02 cm^−1^), and low diffuse reflectance (0.17%), making it suitable for long-range signal propagation. Compared to alternative materials like 3D-printed resins, which exhibit higher optical losses, PMMA allows us to benchmark the performance potential of embedded ToF-based FTIR sensors.

Our implementation supports both flat and curved configurations and provides insight into several key factors: signal decay as a function of distance, effects of axial occlusion, sensitivity to ambient IR interference, and surface shape response. These findings help define the operational boundaries and practical design considerations of future FTIR–ToF interfaces, paving the way toward more compact, scalable, and intelligent tactile surfaces.

Material selection plays a critical role in the performance of optical touch sensing systems, particularly in hybrid configurations such as FTIR–ToF interfaces. Parameters, including refractive index, absorption coefficient, and surface quality, directly influence light propagation, scattering, and ultimately detection accuracy. Recent work by Aulika et al. [9] presented a comprehensive baseline evaluation of various polymer and glass materials for Direct ToF LiDAR tactile sensing, quantifying the impact of optical properties on detection range and signal quality. Such baseline characterization not only informs optimal material choice for specific applications but also provides a framework for predicting system performance during early design stages. The insights from this study are directly applicable to FTIR–ToF touch interfaces, where consistent optical behavior of the waveguide is essential for achieving stable multi-touch detection.

### 1.1. Total Internal Reflection (TIR)

Total Internal Reflection (TIR) is a foundational optical phenomenon occurring when light transitions from a medium with a higher refractive index ($n_{1}$) to a lower one ($n_{2}$), such as from PMMA ($n_{1}\approx 1.49$) to air ($n_{2}\approx 1.00$). When the angle of incidence exceeds the critical angle, no transmission occurs, and the light is completely reflected back into the original medium. The critical angle $\theta_{c}$ is given by Snell’s Law defined below:

(1) $$\theta_{c}=\sin^{-1}\left(\frac{n_{2}}{n_{1}}\right)$$

**Figure 1:**
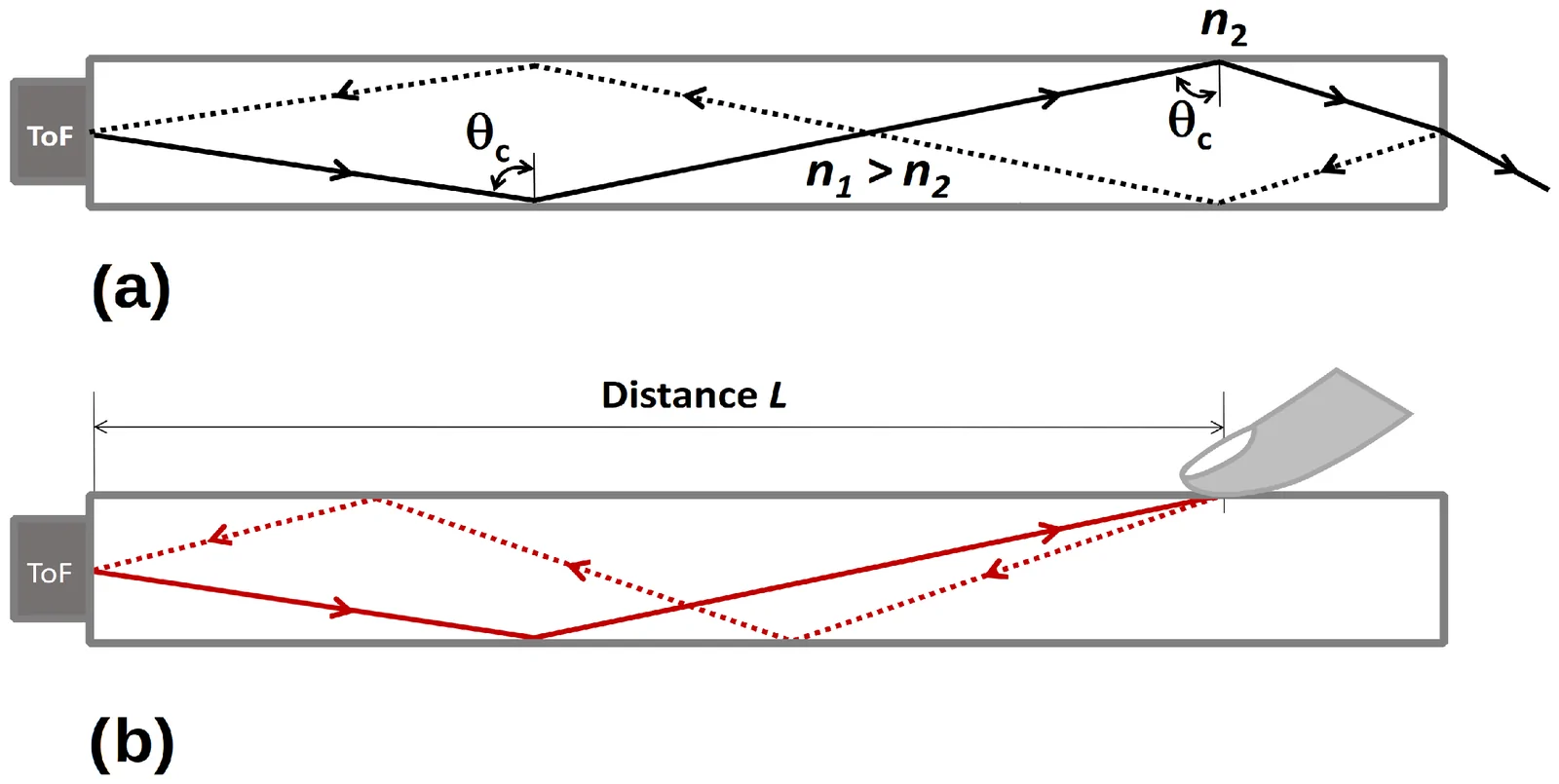
Illustration of TIR and FTIR mechanisms in a waveguide-based optical tactile sensor. (**a**) Light propagation under total internal reflection (TIR) when the incidence angle is greater than the critical angle, θc. (**b**) Touch-induced scattering in frustrated total internal reflection (FTIR), showing how contact interrupts guided light.

This mechanism allows light to propagate through solid transparent materials with minimal loss, enabling applications in waveguides, fiber optics, and optical touch sensing (Figure 1a). In tactile sensing, infrared (IR) light injected into the waveguide via edge-mounted emitters remains confined within the medium through TIR—until it is locally disrupted by physical contact. PMMA is particularly well suited for this due to its optical clarity, low absorption, and ability to maintain TIR even in curved geometries [10,11].

### 1.2. Frustrated Total Internal Reflection (FTIR)

FTIR occurs when the conditions for TIR are met, but a third medium with a higher refractive index than the surrounding environment—such as human skin, oil, or a compliant layer—approaches the interface within the evanescent field range (typically sub-micron). This close proximity allows a portion of the trapped light to couple out via tunneling, disrupting total reflection and scattering the light outward (Figure 1b).

First demonstrated in multi-touch systems by Han [1], FTIR has since evolved into practical designs such as FLATIR [3] and interactive holographic displays [5]. Detection is typically achieved using IR cameras or photodiodes positioned beneath or beside the panel. Contact points appear as bright spots where the light is scattered, and their coordinates are reconstructed through vision-based processing [4,12].

Recent approaches like OptoSkin eliminate external cameras entirely by embedding ToF sensors directly into the waveguide. These sensors measure the time of flight of scattered photons caused by surface deformation or contact [8], enabling not just touch detection but also pressure estimation—particularly in soft waveguide materials. This method facilitates 3D-sensitive, force-aware tactile interfaces and supports curved or conformal surfaces using elastomers, photopolymers, or PMMA waveguides [8].

The fusion of FTIR and ToF unlocks new possibilities for scalable, electrode-free, camera-free tactile systems, particularly suited for robotic skins, wearables, and large-area interfaces where robustness and modularity are key.

## 2. Materials and Methods

### 2.1. Optical Measurements

The variable angle J. A. Woollam Spectroscopic Ellipsometer (SE) RC2–XI (J. A. Woollam Co., Inc., Lincoln, NE, USA) was employed to acquire refractive coefficient (n) and extinction coefficient (k) data across the spectral range of 210 to 1690 nm, equivalent to 0.7 to 5.9 eV. Ellipsometry spectra were collected at incident angles ranging from 50° to 65°, with a 5° increment. Analysis of the experimental SE data was conducted using the CompleteEASE^®^ software version 6.66 (J. A. Woollam Co., Inc., Lincoln, NE, USA), employing model-based regression analyses. The dispersion curves of optical constants were modeled utilizing the Tauc–Lorentz oscillator parameterization, while the surface roughness was characterized using the Bruggeman Effective Medium Approximation (EMA).

Transmittance measurements were performed using a double-beam Cary 7000 spectrophotometer (Agilent Technologies Inc., Santa Clara, CA, USA), operating in the 175–3300 nm spectral range. This device is equipped with tungsten halogen (Vis-NIR) and deuterium (UV) lamps. For detection, an R928 PMT in the UV-Vis range and a cooled PbSmart PbS detector in the NIR range were used. The system’s limiting resolution ranges from 0.048 nm (UV-Vis) to 0.2 nm (NIR).

### 2.2. PMMA Preparation

For optimal performance, critical parameters include the surface roughness, dispersion coefficient, and absorption coefficient of the PMMA material at the operating wavelength of the sensor. Equally crucial is the effective coupling between the sensor and the light guide to minimize transition losses of laser radiation from one medium to another. In the context of this technology, enhancing the light guide’s material interaction efficiency has been achieved through precise laser processing.

**Figure 2:**
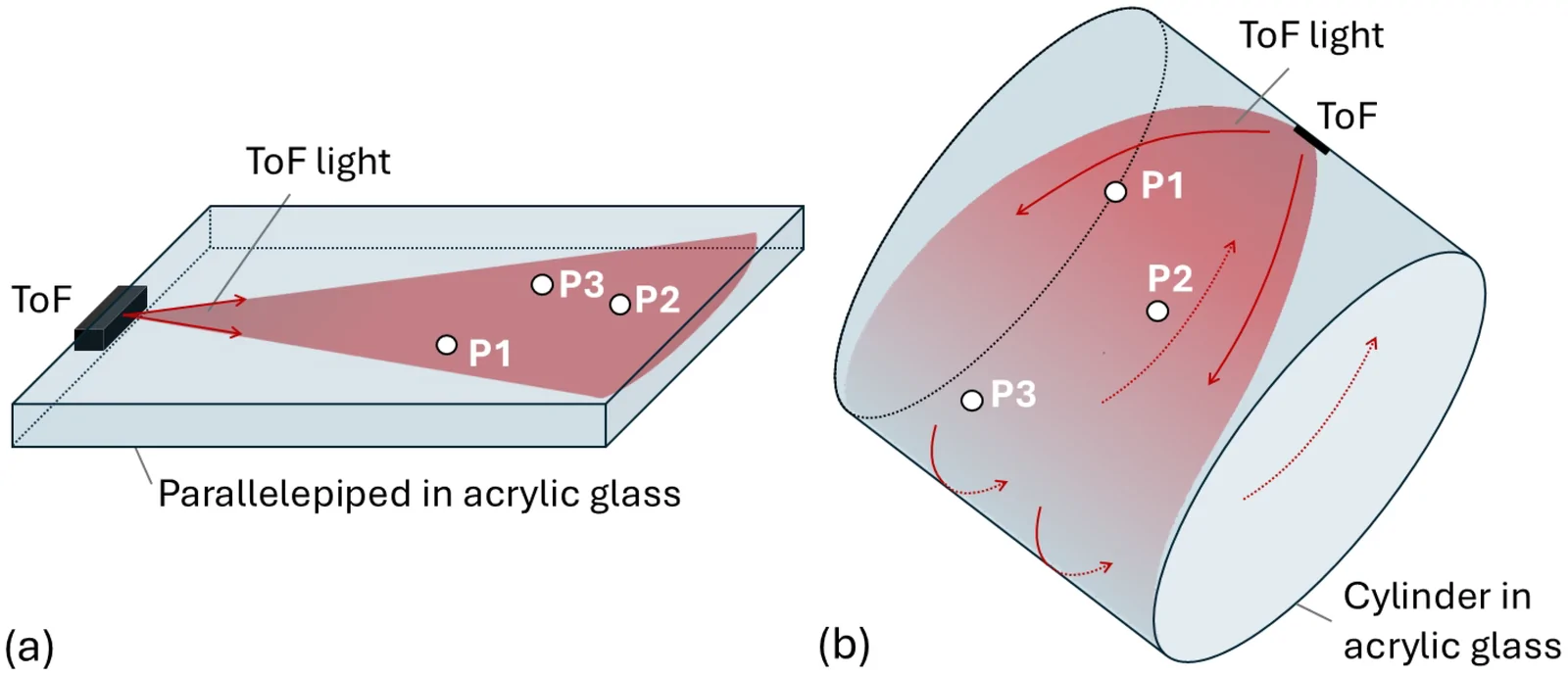
Schematic representation of an acrylic light guide used for ToF sensing. (**a**) Flat configuration: A 2 mm thick parallelepiped-shaped acrylic guide with dimensions 300 mm (length) × 200 mm (depth) integrates a ToF sensor positioned at one edge. The red cone illustrates the emitted and reflected light propagation within the waveguide by total internal reflection. Contact points P1, P2, and P3 correspond to surface touch locations at distances of 176.64 mm, 240.3 mm, and 201.32 mm from the ToF sensor, respectively. (**b**) Curved configuration: The same light guide is formed into a cylindrical arc to demonstrate signal behavior along a bent surface. Light continues to propagate internally and reflects from contact-induced scattering events at P1–P3. Beveled corners are employed to optimize light confinement and improve signal consistency across the sensing area.

**Figure 3:**
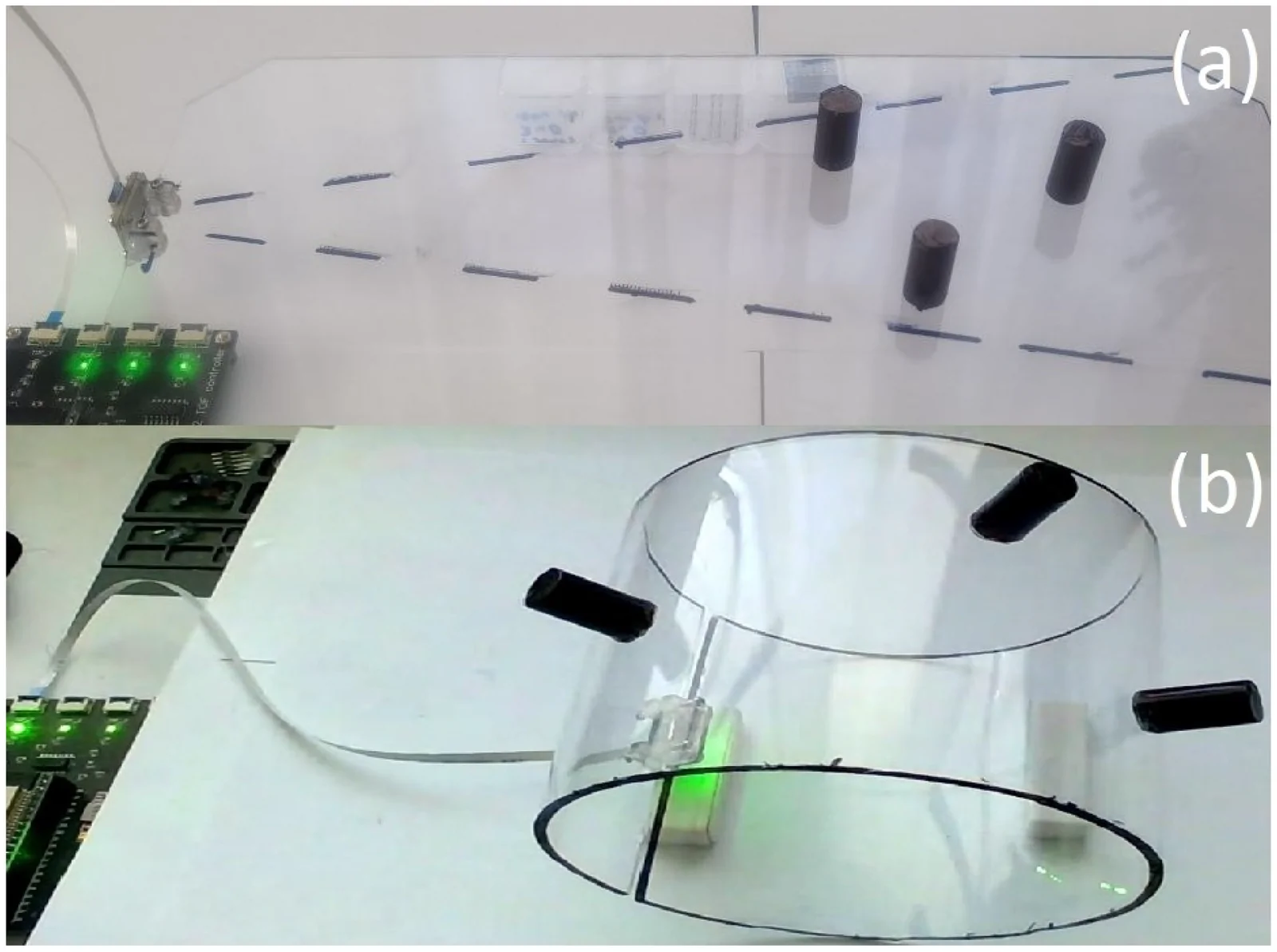
Photographs of the experimental setup designed to investigate the propagation of ToF sensor radiation within a PMMA (poly(methyl methacrylate)) light guiding sheet. (**a**) Flat PMMA light guide (300 mm long) with three black test cylinders placed on the surface within the FoV of the ToF sensor (outlined by a dashed line). (**b**) Cylindrical PMMA light guide with the same three cylinders positioned within the sensor’s FoV for comparison.

A sheet of cast acrylic glass, specifically poly(methyl methacrylate) (PMMA) with the CAS number 9011-14-7, measuring 200 × 300 × 2 mm (see Figure 2a), was utilized as a light guide in the experiments. The edges of this material were mechanically polished to achieve maximum transparency, which is critical to minimize light scattering at the air/PMMA interface. The same acrylic material was also used in a shape of cylinder with a height of 96 mm and a diameter of 107 mm (Figure 2b). A cut was made on the surface of the cylinder to install the TOF sensor. To reduce light interference from external background illumination and signal reflections from the edges of the material, the edges were treated with light-absorbing paint, as shown in Figure 3a,b. Following the preparation of the light guide, the TOF sensor was installed and configured to interact with this setup. Upon successful configuration, all sensor zones must display the distance to the far edge of the light guide multiplied by the optical refractive index of PMMA for the operating wavelength of the sensor, providing an accurate representation of the light path within the guide.

### 2.3. Test Objects

Three cylindrical test samples, each made of soft silicone rubber with a diameter of 10 mm, were placed at different locations on the surface of the acrylic glass within the FoV of the ToF sensor according to the scheme represented in Figure 2 and Figure 3.

### 2.4. Electronic Hardware

The ToF sensor TMF8828 (ams-OSRAM AG, Premstätten, Austria) was selected for its high compatibility with the optical and spatial requirements of the proposed FTIR–ToF system. The TMF8828 integrates a multi-zone direct ToF measurement capability, offering up to 64 detection zones with millimeter range precision, which is critical for accurate tactile and proximity sensing over extended surfaces. Additionally, its compact footprint and wide FoV make it particularly well suited for integration into planar and curved optical waveguide structures without introducing significant optical distortion or bulk.

Sensor data readout and configuration were performed via the I^2^C communication protocol, operating at clock speeds of up to 1 MHz. An ESP32-S3 microcontroller unit (MCU) was selected as the host platform, offering both the computational resources necessary for real-time preprocessing and wireless communication capabilities (Wi-Fi and Bluetooth) for data transmission to a remote host computer. The MCU setup is scalable, supporting up to four TMF8828 sensors simultaneously, enabling modular expansion of the tactile sensing surface if needed.

Each TMF8828 sensor is mounted on an individual printed circuit board (PCB), which interfaces with the ESP32-S3 via flexible printed circuits (FPCs). The use of FPCs allows for adjustable sensor positioning along the PMMA light guide edges to maximize optical coupling efficiency while minimizing mechanical stress on the system.

**Figure 4:**
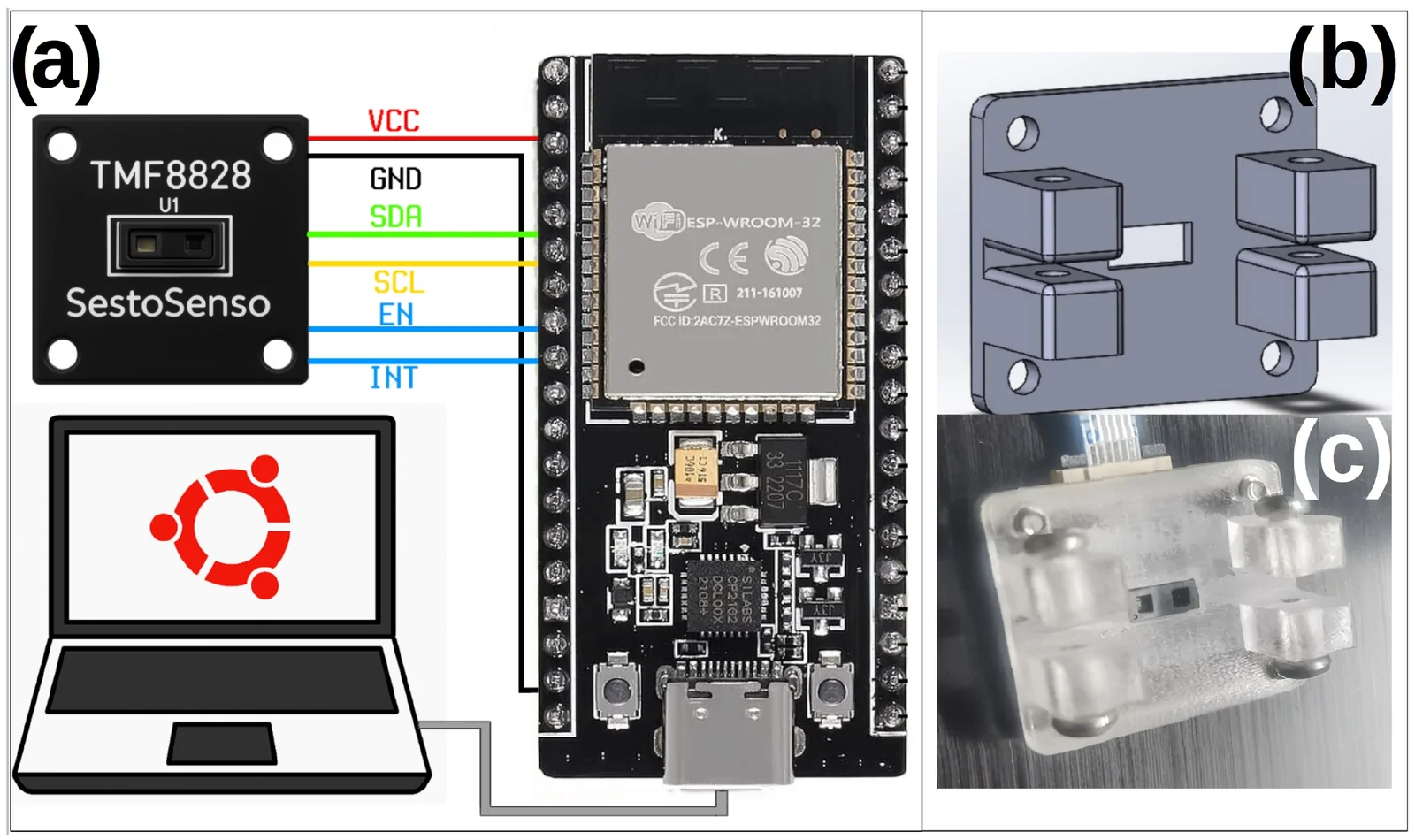
Overview of the electronic and mechanical integration of the TMF8828 ToF sensor module with an ESP32 microcontroller platform. (**a**) Electrical schematic showing the I^2^C communication lines (SDA, SCL), power (VCC, GND), and control signals (EN, INT) between the TMF8828 sensor and ESP32 MCU, with data transmission to a host computer. (**b**) CAD rendering of the custom-designed optical adapter for aligning the sensor with the light guide. (**c**) Photograph of the fabricated adapter made of transparent polymer material, demonstrating the physical installation of the TMF8828 sensor within the optical assembly.

Precise optical alignment between the sensor modules and the PMMA waveguide was achieved through the use of custom 3D-printed adapters, as illustrated in Figure 4b). These adapters are specifically designed to match the optical path requirements and the 2 mm thickness of the PMMA material. They ensure consistent positioning of the ToF sensor’s emitter and receiver relative to the light guide boundary, thereby minimizing coupling losses and ensuring high repeatability in sensor calibration.

### 2.5. Sensor Data Acquisition and Reconstruction

**Figure 5:**
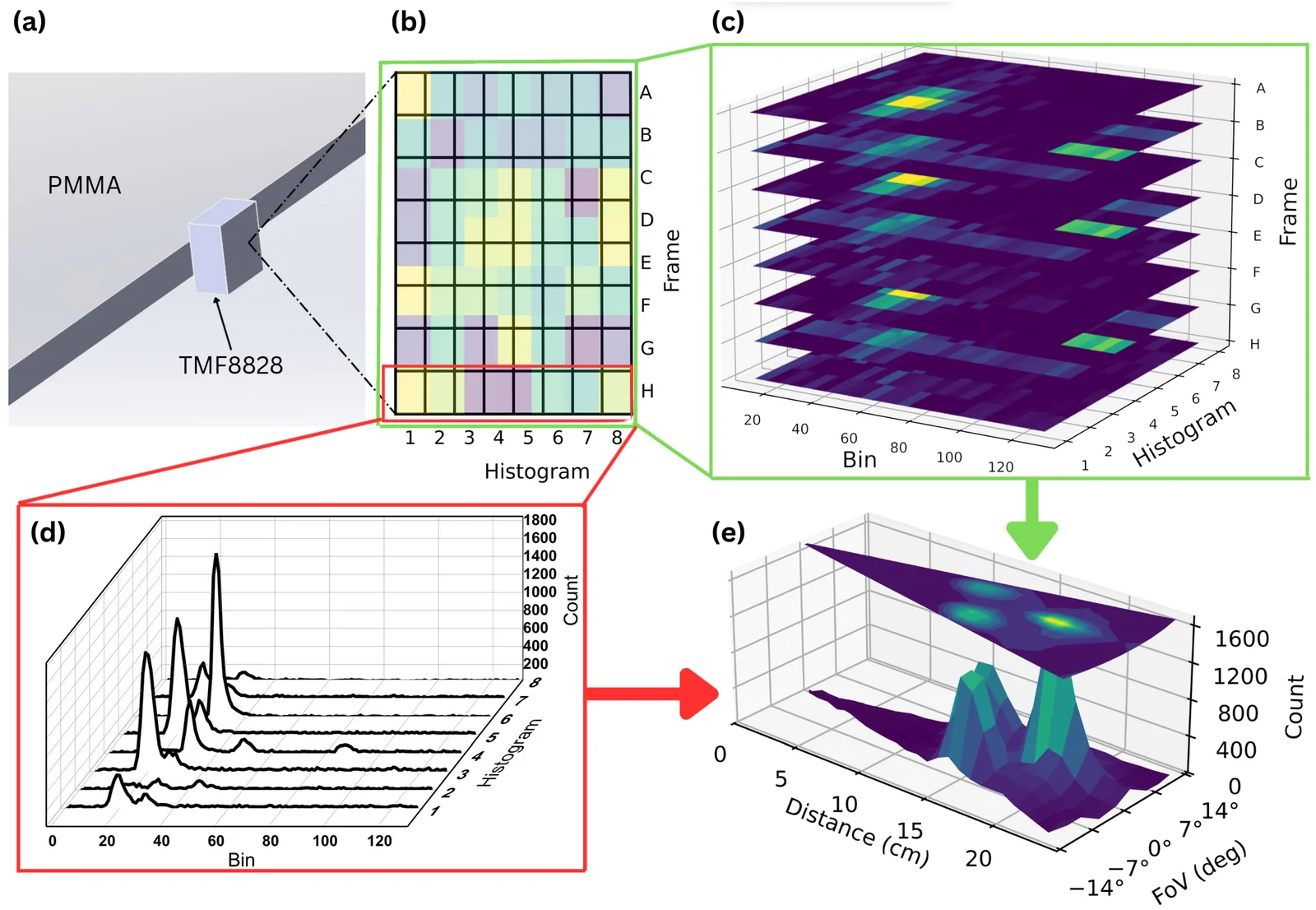
TMF8828 data acquisition and preprocessing workflow in the FTIR–ToF configuration. (**a**) Coupling of TMF8828 sensor with PMMA waveguide. (**b**) Multi-zone histogram arrangement into eight horizontal frames (A–H). (**c**) Reconstructed 3D histogram volume from reordered SPAD data. (**d**) Individual histogram photon count distributions as a function of TDC bin index. (**e**) Summed and ROI-selected frame data used for FTIR touch detection.

The TMF8828 sensor operates in an 8 × 8 multi-zone mode, generating 240 raw histograms per snapshot. Owing to its 24-bit Time-to-Digital Converter (TDC) resolution, data are transmitted via the I^2^C interface as three 8-bit chunks, forming a 28,800-byte packet. After acquisition, the host MCU reconstructs these data into an 80 × 128 array, which is subsequently reordered according to the sensor’s Single-Photon Avalanche Diode (SPAD) mask configuration (see AMS OSRAM documentation [13]). Following the exclusion of 16 service histograms, the final 64 × 128 dataset was arranged into eight horizontal frames (A–H), each containing eight histograms (Figure 5a–d).

To reconstruct a complete 8 × 8 frame in TMF8828 mode, four time-multiplexed sub-captures are required, each containing 10 histograms. Within each sub-capture, histogram 0 (reference) and histogram 9 (unused) are discarded, yielding 8 × 4 = 32 useful histograms per frame. This reconstruction step requires that each histogram be placed into the correct spatial position based on the active SPAD mask and sub-capture order. A supplementary Python script is provided to illustrate the reconstruction workflow: The script parses raw histogram lines, extracts a user-defined bin-range region of interest (ROI), reorders the 32 histograms into an 8 × 8 spatial layout, and produces both a heatmap and a horizontal polar plot of the reconstructed signal distribution. The inclusion of this script is intended to provide methodological transparency and enable reproduction of the processing pipeline by other researchers.

For FTIR applications, all columns within the ROI are summed across bins to enhance touch-induced scattering signals (Figure 5e) while preserving horizontal spatial resolution. In soft polymer waveguides (e.g., silicone), histogram deformation patterns across all eight frames can be analyzed to estimate local pressure, as demonstrated in our earlier work [7]. The TMF8828 supports both long-range (up to 5 m) and short-range (up to 1 m, higher accuracy) operating modes; the latter was selected for this study to maximize touch detection precision in the targeted application scenario.

## 3. Results and Discussion

### 3.1. Optical Properties

**Figure 6:**
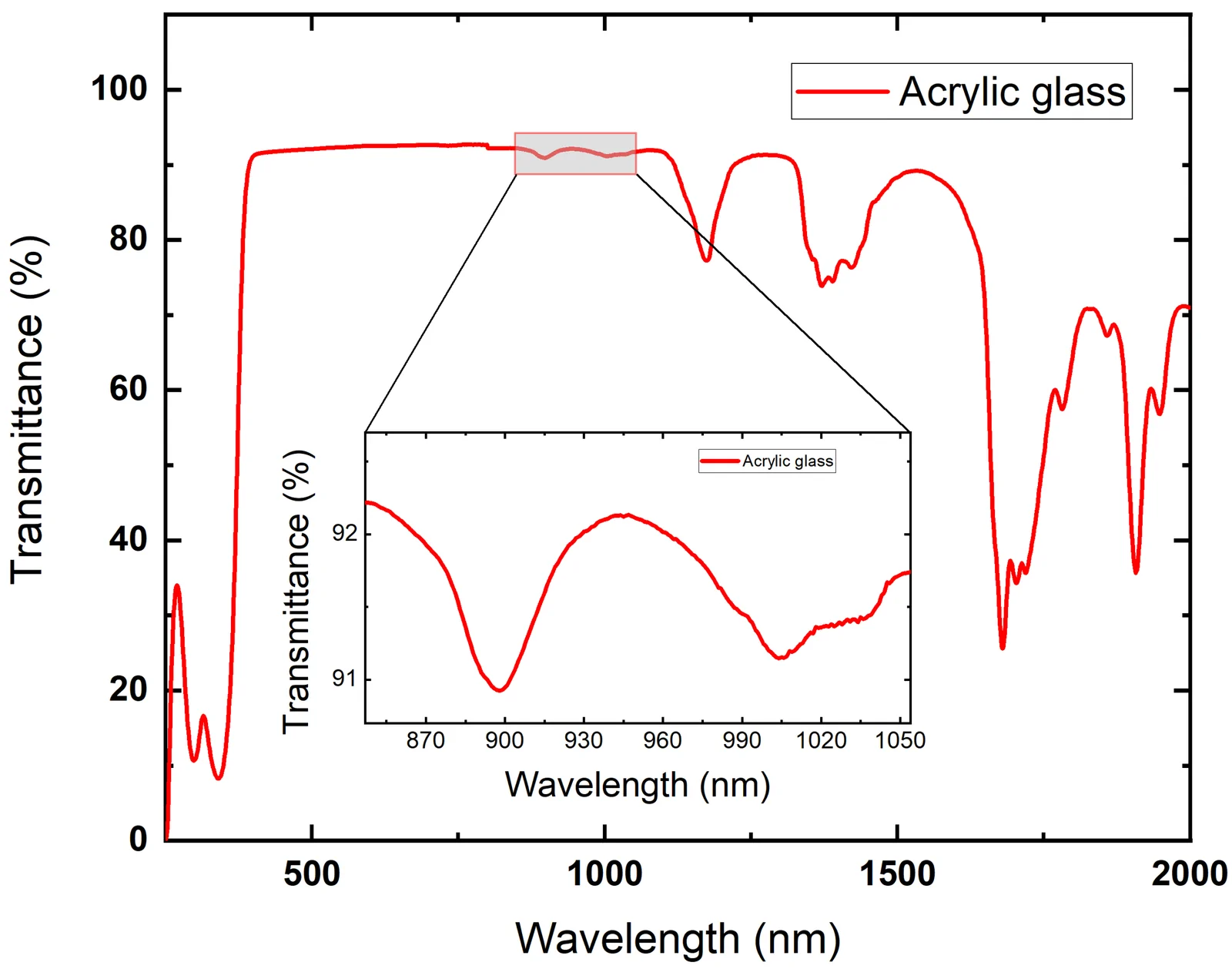
The results of the transmission spectrum measurement for 2 mm thick acrylic glass. At the wavelength of 940 nm, the transmittance level reached 92.11%.

The transmittance spectrum Figure 6 for acrylic glass is of particular relevance when considering its application as a light guide for ToF sensors, which commonly utilize 940 nm laser radiation. The graph reveals that acrylic glass exhibits exceptional optical clarity in the visible spectrum, with transmittance rates approaching 100% up to about 1000 nm, indicating minimal light loss due to absorption or scattering. This characteristic ensures that the acrylic material can effectively channel visible light. However, the performance of the acrylic glass as a light guide is most critical in the near-infrared region, especially around the 940 nm wavelength used by ToF sensors. The inset zoom within the graph specifically highlights the behavior of acrylic glass around this wavelength, where it maintained a transmittance percentage within the range of 91% to 93%. The consistency of transmittance near the 940 nm range suggests that acrylic glass can serve as an efficient light guide for the 940 nm laser radiation employed in ToF sensors, enabling precise and accurate distance measurements by facilitating the transmission of light with minimal attenuation. The detailed spectrum also demonstrates the material’s complex interaction with infrared light, presenting significant absorption features beyond the operational wavelength of ToF sensors, which could be advantageous in preventing interference from other infrared sources.

In Figure 6, the optical transmittance of acrylic glass in the 250–2000 nm spectral range is shown. Acrylic glass is highly transparent in the 400–1125 nm range, where the optical transmittance exceeds 90%. For longer wavelengths, spectral minima occurred due to absorption, and optical transmittance decreased abruptly for shorter wavelengths (<400 nm); the material became opaque below 250 nm. In the 900–1000 nm spectral range (Figure 6, inset), a local transmittance maximum at 945 nm was observed, reaching 92.15%; the transmittance at 940 nm was 92.10%. The measurement error was 0.02%. Thus, the optical properties of the material are well suited for the development of a TOF sensor operating at 940 nm.

### 3.2. Signal Processing

#### 3.2.1. Signal Decay Characterization

**Figure 7:**
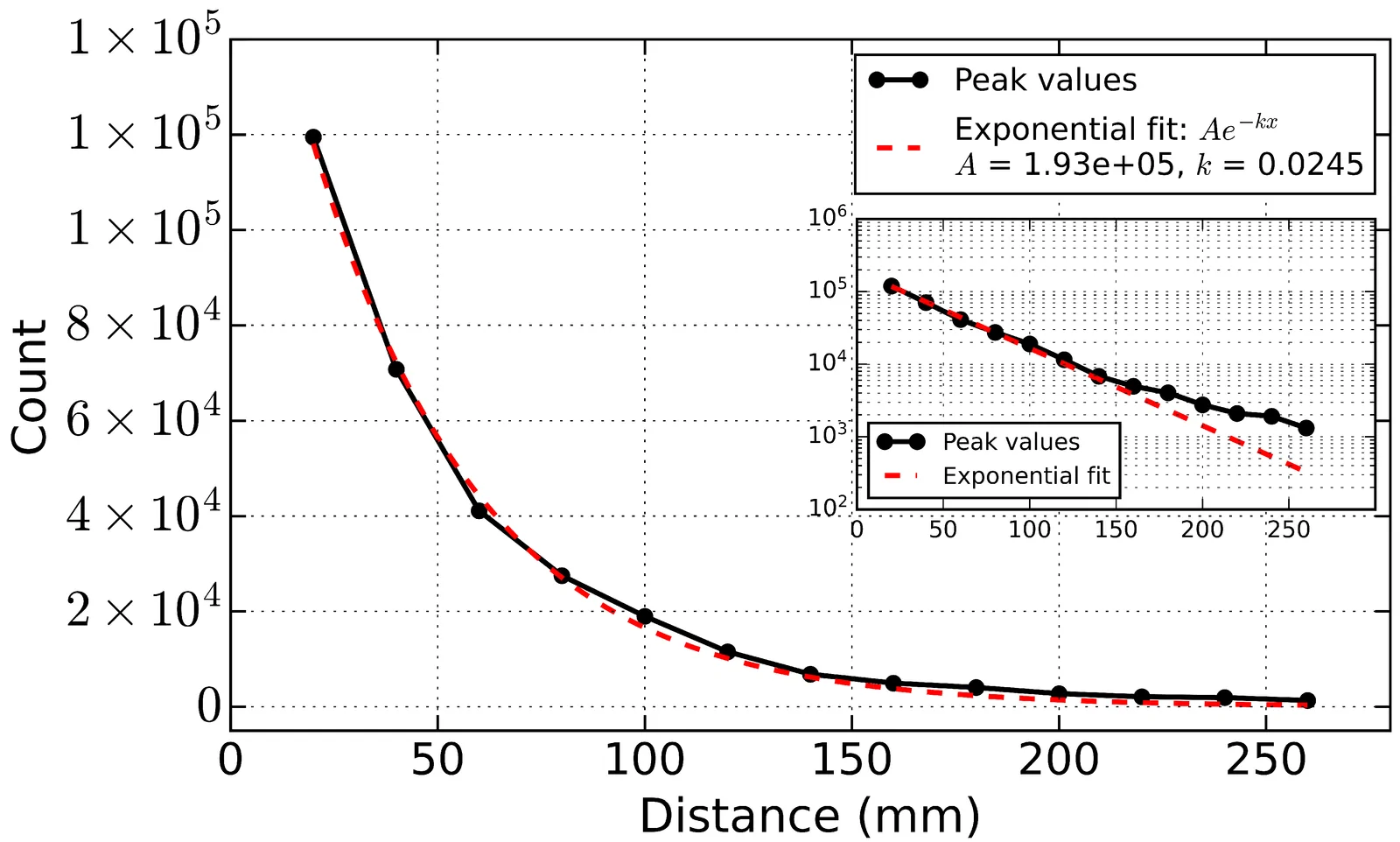
Exponential decay of peak signal intensity as a function of distance from the ToF sensor for silicone test objects. Black markers indicate measured values, and the red dashed line represents the fitted exponential model $Ae^{-kx}$, with $A\;=\;1.93\;\times \;10^{5}$ and $k\;=\;0.0245\;\;{\mathrm{mm}}^{-1}$. The inset shows the same data on a logarithmic scale to highlight the linearity of the decay trend. No significant differences were observed between flat and curved PMMA configurations.

To quantitatively assess the attenuation of the scattered light signal within the PMMA light guide, a systematic experiment was conducted using silicone test objects positioned at distances ranging from 20 mm to 260 mm in 20 mm increments. Peak signal values were extracted from the ToF sensor readings at each position. As illustrated in Figure 7, the signal intensity exhibits a clear exponential decay with increasing distance from the sensor, conforming to the model $Ae^{-kx}$, where the fitted parameters are $A=1.93\times 10^{5}$ and $k=0.0245\;{\mathrm{mm}}^{-1}$. The strong agreement between the experimental data and the exponential fit indicates a consistent attenuation profile governed primarily by the intrinsic optical properties of the PMMA and the quality of sensor coupling. Comparative measurements performed on flat and curved PMMA configurations revealed no statistically significant differences in the decay trend within the experimental margin of error, suggesting that waveguide geometry does not critically affect attenuation behavior under controlled conditions. This result underscores the flexibility of the proposed sensing architecture for use in both planar and conformal tactile surfaces without the need for recalibration of signal thresholds.

#### 3.2.2. Axial Occlusion and Signal Interference

**Figure 8:**
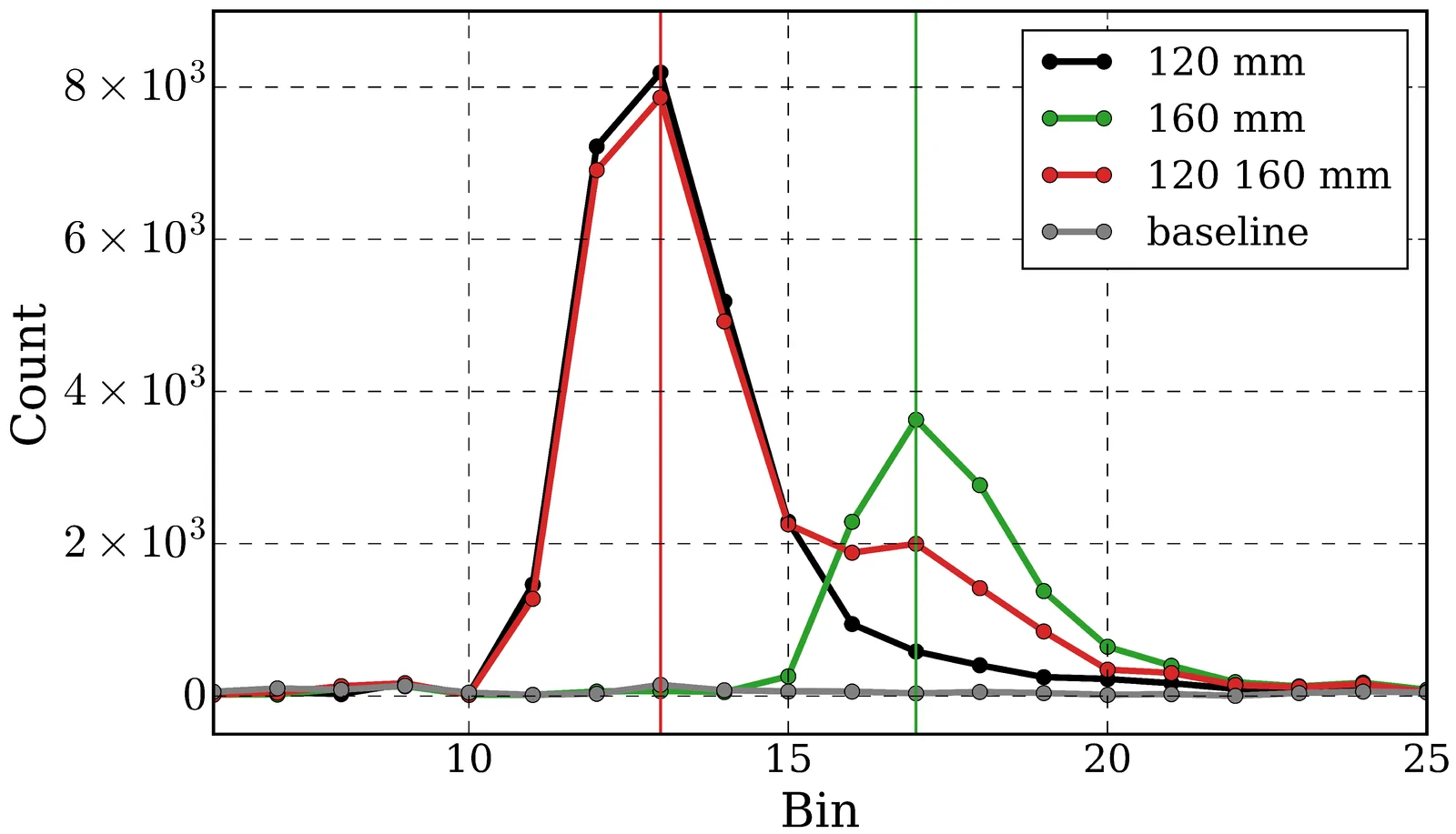
Histogram comparison showing the effect of axial occlusion within the PMMA waveguide. Black and green curves represent isolated contacts at 120 mm and 160 mm, respectively. The red curve shows the combined signal when both contacts are present, with the 120 mm object partially occluding the 160 mm position. The gray curve shows the baseline signal. Peak amplitude at 160 mm decreased by ∼45% due to occlusion, while the 120 mm signal dropped only ∼4%.

To study how overlapping touch points influence signal intensity within the PMMA waveguide, a sequential measurement experiment was performed. A silicone test object was first placed at a distance of 160 mm from the ToF sensor, and the corresponding histogram response was recorded. Then, a second identical object was introduced along the same optical axis at 120 mm—effectively occluding the forward scattering path from the original contact point. As shown in Figure 8, the signal at the 160 mm position (green curve) was notably diminished when the 120 mm object was present (red curve), while the signal for the nearer object remained dominant and relatively unaffected.

Quantitatively, the peak amplitude of the 160 mm signal in isolation was 3630 counts, which dropped to 2000 counts in the occluded case—a relative decrease of approximately 44.9%. In contrast, the signal at 120 mm exhibited only a modest reduction from 8191 to 7862 counts (approximately 4.0%) when the 160 mm object was added behind it. This asymmetry confirms that the foreground object strongly interferes with detection of the background signal, while the reverse effect is negligible.

These results highlight the ToF system’s depth sensitivity and confirm its ability to resolve simultaneous contacts separated along the optical axis. The observed attenuation also suggests that histogram-based analysis may enable touch depth estimation or contact ordering in future applications, particularly when dealing with overlapping input events in robotic skin or high-density touch arrays.

#### 3.2.3. Interference Immunity to External Infrared Sources

To evaluate the robustness of the FTIR–ToF system against external infrared interference, a controlled experiment was conducted using a standard 3 mm IR LED (940 nm) placed at the opposite edge of the PMMA light guide along the same optical axis as the ToF sensor. A silicone test object was fixed at 120 mm from the sensor, and ToF histograms were recorded under varying LED drive currents: 5 mA to 35 mA in 5 mA steps. A baseline reference was first acquired with the LED turned off.

**Figure 9:**
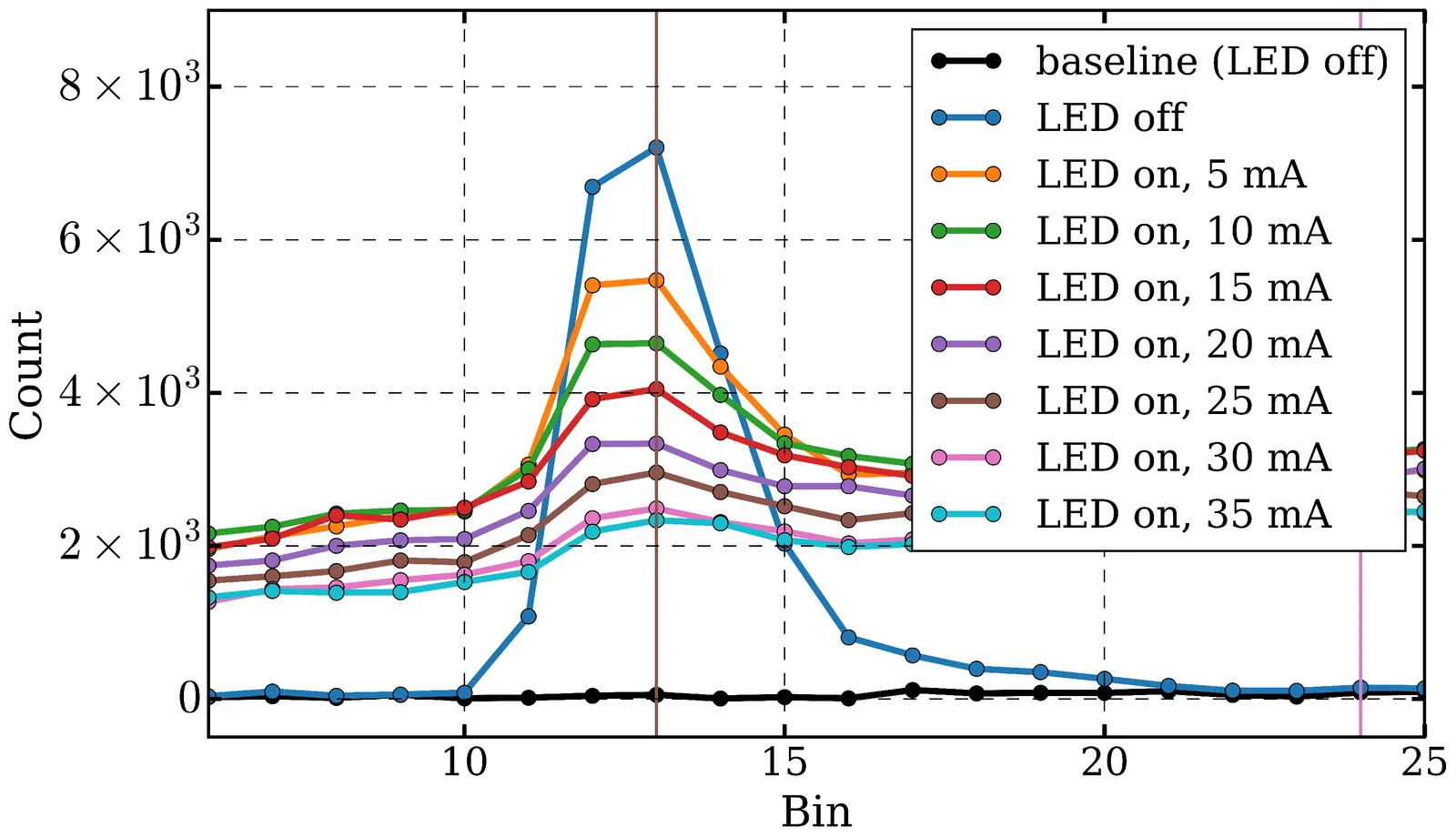
Effect of external 940 nm infrared interference on ToF signal amplitude. A silicone test object was positioned at 120 mm from the sensor. The baseline signal (LED off) peaked at approximately 7800 counts. As the LED current increased from 5 mA to 35 mA, signal amplitude steadily declined, dropping by ∼30% at 20 mA and by over 70% at 35 mA. The system maintained detection capability, highlighting its interference tolerance.

As shown in Figure 9, the baseline signal (LED off) reached a peak of approximately 7800 counts. When the LED was activated at 5 mA, the signal immediately showed a noticeable decrease, indicating the onset of optical interference within the waveguide. As the LED current increased, the signal amplitude continued to decline progressively. By 20 mA, the peak response dropped by roughly 30% compared to the baseline. At 35 mA, the signal fell to less than 25% of the baseline, confirming a strong saturation or crosstalk effect due to excessive background IR.

Despite this degradation, the system maintained a discernible response across all interference levels, and signal extinction was never complete. This demonstrates a degree of resilience in the sensor design, particularly in terms of dynamic range and tolerance to optical noise at the ToF’s operating wavelength. These findings support the feasibility of deploying FTIR–ToF systems in uncontrolled lighting conditions, especially when combined with edge shielding, modulation filtering, or adaptive thresholding strategies.

**Figure 10:**
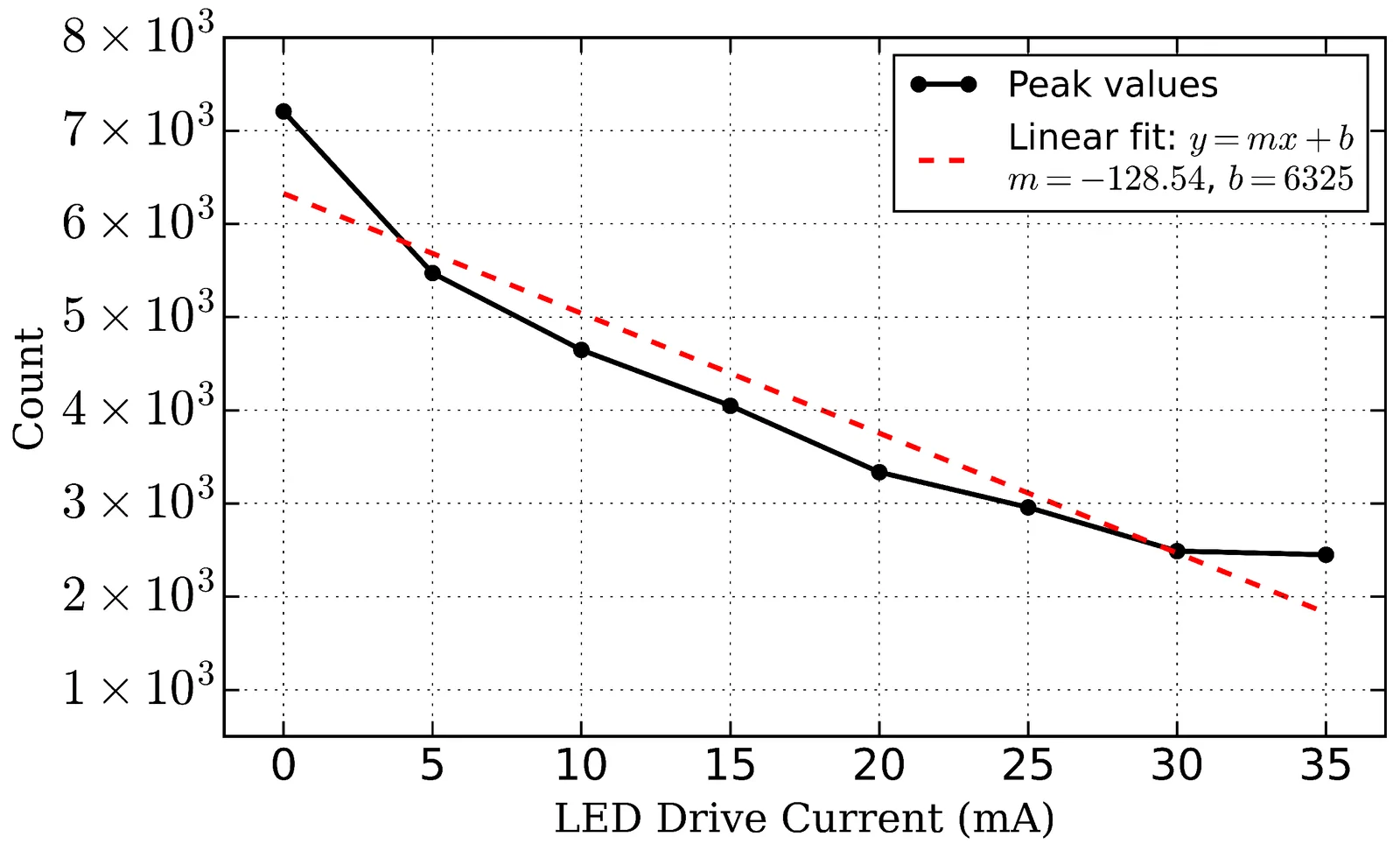
Measured peak signal amplitudes at a fixed distance of 120 mm as a function of LED interference current from 0 to 35 mA. A linear trend is observed, described by the fit y=mx+b, with slope m=−128.54 and intercept b=6325. The results highlight the degradation of signal integrity due to increasing IR interference.

To assess the susceptibility of the signal detection mechanism to external infrared (IR) interference, a series of measurements was conducted at a fixed object distance of 120 mm while varying the drive current of an external IR LED source from 0 to 35 mA. As shown in Figure 10, the recorded peak signal amplitude decreased nearly linearly with increasing IR intensity. The observed decay follows a linear trend with a slope of approximately −128.5counts/mA. Over the full range, this corresponds to a total signal reduction of roughly 66%, indicating significant attenuation under high ambient IR load. These results demonstrate the importance of either shielding the sensor or implementing adaptive IR rejection techniques in practical applications.

### 3.3. Baseline Study for Material Quality Assessment

**Figure 11:**
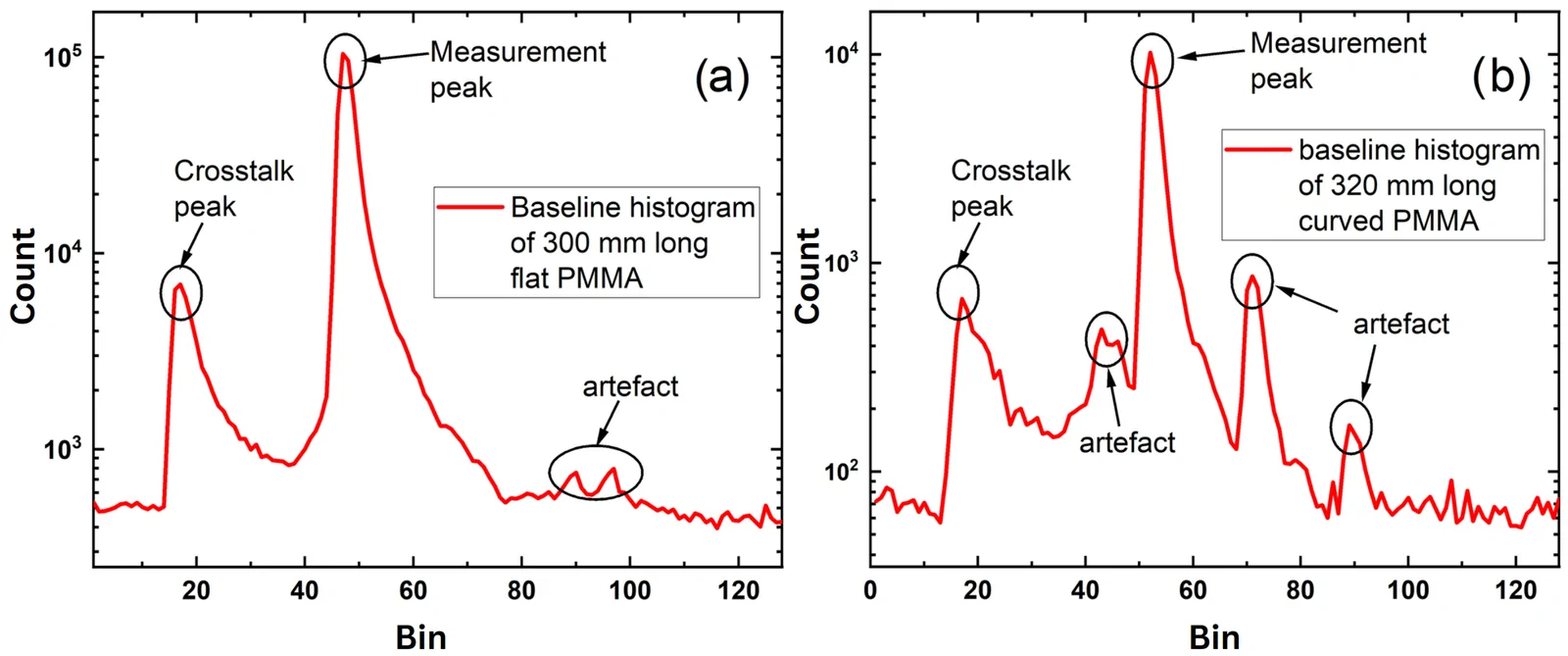
Histograms of ToF signal interactions with PMMA light guides. (**a**) shows the response from the flat PMMA light guide, while (**b**) depicts the response from the cylindrical PMMA light guide.

The histograms in Figure 11a,b illustrate the interaction of ToF signals with two different acrylic glass shapes, flat and cylindrical (curved), each 300 mm long, with the sensor positioned at the edge of the light guide. The first peak at bin 15 in both cases corresponds to a superposition of crosstalk and a reflection from the near edge of the light guide, while the second peak at bin 47 represents a reflection from the far edge. The region of interest (ROI), spanning 32 bins, covers the length of the light guide, providing a linear resolution of 9.4 mm per bin in short-range mode. Artifacts observed in both histograms, particularly pronounced in the curved glass (Figure 11b), are attributed to material inhomogeneities and coupling inefficiencies between the sensor and the light guide, which result in scattered light and signal degradation. These artifacts reduce the signal-to-noise ratio, especially in the cylindrical configuration, leading to a noisier and less defined signal compared to the flat PMMA. It should be noted that signal artefacts are not inherently determined by the geometric shape of the light guide (e.g., straight, curved, or irregular). Instead, their occurrence and characteristics depend on factors such as material homogeneity, surface finish, coupling efficiency, and ambient light conditions. These artefacts can be mitigated through baseline subtraction during signal processing, which removes static background components from the measured signal. Baseline subtraction may be applied once during calibration or continuously in real time, improving reconstruction accuracy across different light guide geometries. This baseline study approach can also be applied for preliminary evaluation of the optical quality of a selected light guide, as demonstrated by Aulika et al. [9], where systematic characterization of refractive index, absorption, and scattering provided predictive insight into sensing performance.

The insights gained from this baseline characterization directly inform the subsequent touch point reconstruction process, enabling more accurate mapping by accounting for material-induced signal distortions and optimizing the parameters of the reconstruction workflow.

#### 3.3.1. Touch Point Reconstruction and Signal Mapping

**Figure 12:**
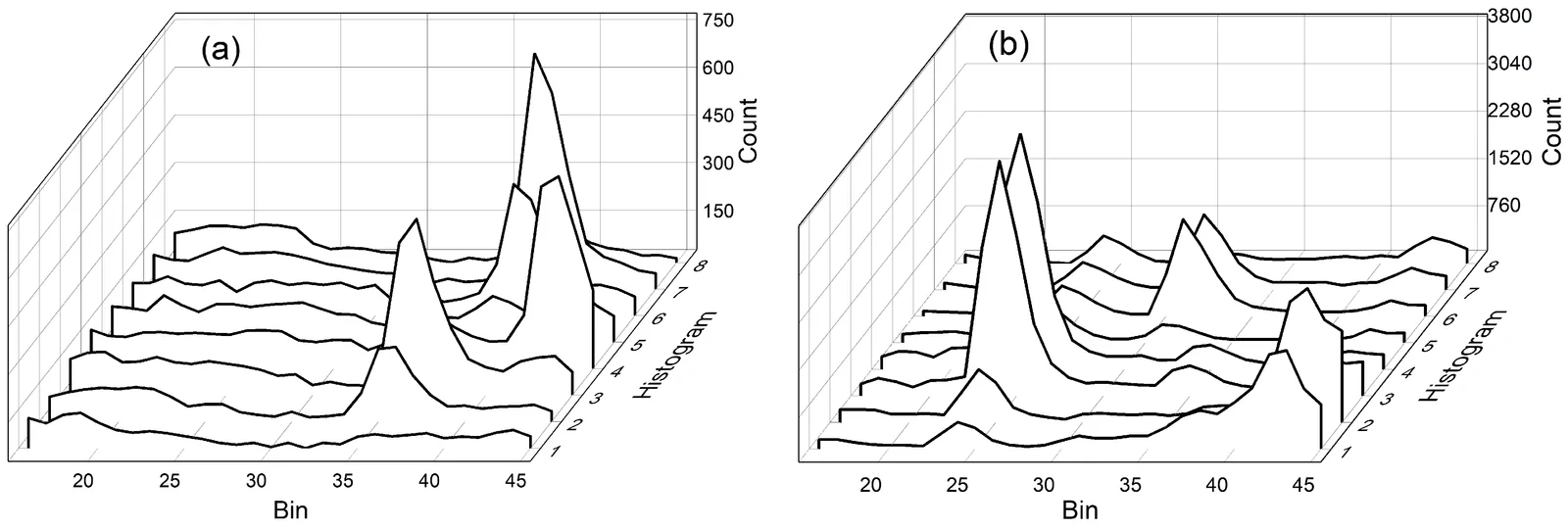
Example frames of summed histograms obtained with three test objects attached to a PMMA light guide in two configurations: (**a**) flat geometry and (**b**) curved geometry. Each plot represents photon count distributions across histogram bins for the eight angular frames (A–H), illustrating the influence of light guide shape on signal patterns.

**Figure 13:**
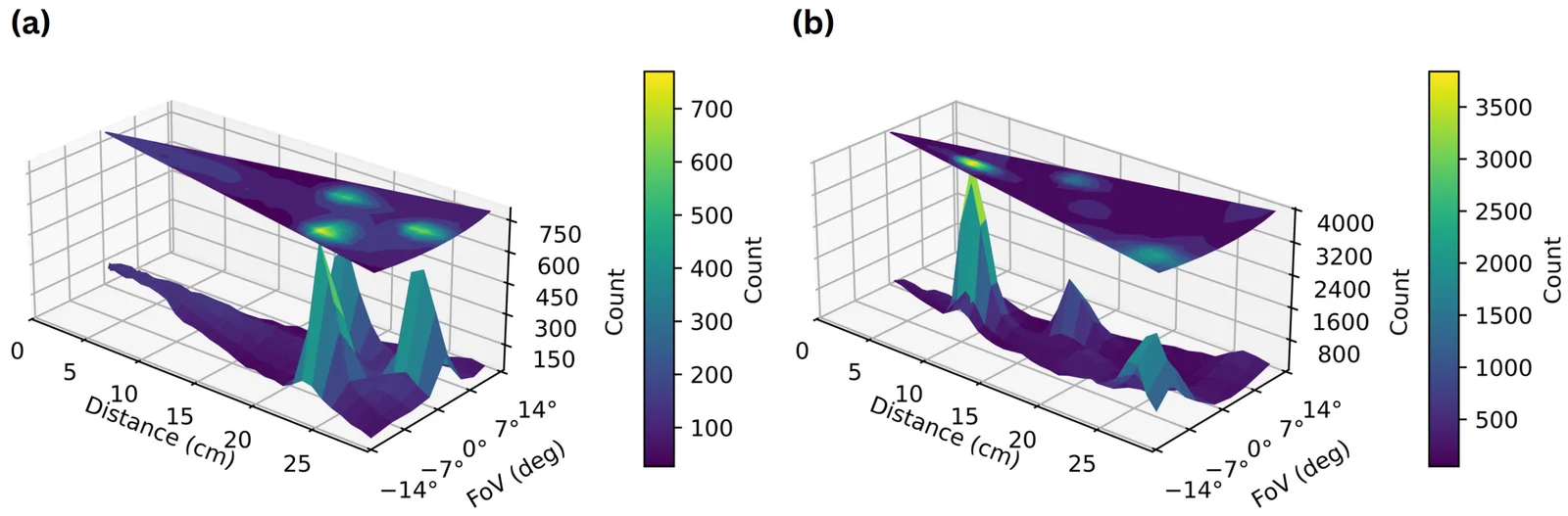
Figure showcases a three-dimensional representation of a multi-touch signal, reconstructed in polar coordinates for flat light guide (**a**) and curved (**b**). The x, y, and z axes correspond to spatial dimensions and signal intensity, respectively. The graph highlights the distribution of touch points across the surface of the sensor, with varying signal intensities depicted by the height and color gradients. Reconstruction of the FoV of the single ToF sensor and signatures from simultaneous contacts with the surface at multiple points.

Figure 12 and Figure 13 illustrate the reconstruction of touch points and the mapping of scattering signal intensity for two PMMA light guide configurations: flat and curved. These results were obtained using the TMF8828 ToF sensor operating in 8 × 8 multi-zone mode, generating 240 raw histograms per acquisition. The acquisition and preprocessing steps follow the method outlined in Section 2.5, including histogram reordering according to the SPAD mask configuration, summation of histograms across frames to enhance scattering signatures, and baseline subtraction to remove static artefacts caused by crosstalk, light guide edge reflections, and environmental light interference. This preprocessing significantly improves the signal-to-noise ratio and facilitates the accurate localization of touch-induced scattering peaks.

Figure 12 presents example summed histograms for eight frames (A–H) shown separately for the flat (a) and curved (b) light guide configurations. The *x* axis corresponds to histogram bin number, representing optical path length, and the *y* axis to the histogram index within a single frame. For both geometries, two high-intensity peaks dominated: The first from the near edge of the light guide, and the second from the far edge. Touch interactions produced intermediate peaks within the region of interest (ROI), which spanned approximately 32 bins. This ROI corresponds to a linear spatial resolution of about 9.4 mm per bin in short-range mode. In the curved light guide configuration, the measured signal exhibited more pronounced scattering artefacts and a lower peak-to-noise ratio, being primarily due to reduced coupling efficiency and increased scattering from surface imperfections.

Figure 13 shows the reconstructed polar-coordinate maps of the sensor’s field of view (FoV) for the flat (a) and curved (b) light guides. In these plots, the radial axis represents the distance from the sensor, the angular axis corresponds to the FoV in degrees, and the vertical axis depicts photon count intensity. The color gradient provides an additional measure of intensity variation. These reconstructions make it possible to localize multiple simultaneous touch points across the light guide’s surface, with distinct separation in both radial and angular coordinates. The difference between the flat and curved geometries is clearly visible: while the flat guide maintains sharper, more localized peaks, the curved guide shows broader, less-defined scattering regions, indicating higher levels of optical loss and dispersion.

This approach enables touch localization with a linear resolution of approximately 10 mm and an angular resolution of up to 3.5° for the selected PMMA, depending on the optical and geometric properties of the light guide. The method is geometry-agnostic in principle but requires calibration and baseline subtraction to correct for material inhomogeneity, surface finish variability, and coupling efficiency differences. While the examples presented here focus on PMMA, the same workflow can be adapted for alternative transparent polymers or glass, provided that optical properties such as refractive index, absorption, and scattering are well characterized prior to deployment. Real-time multi-touch operation is demonstrated in Supplementary Video S1 (flat PMMA prototype) and Supplementary Video S2 (curved light guide configuration).

#### 3.3.2. System Accuracy and Scalability

While the TMF8828 ToF sensor nominally achieves 1 mm accuracy in short-range mode via proprietary histogram processing, our unconventional use case (FTIR-coupled detection) requires additional postprocessing to map raw ToF data to physical touch coordinates. Distance values for each zone can be extracted alongside histogram data and used as correction factors, but this necessitates custom algorithms beyond the scope of the present work. Future implementations could leverage such corrections to approach the sensor’s native precision.

Importantly, many public-facing touch applications, such as ATMs or ticket machines, prioritize robustness, cost-efficiency, and ease of maintenance over sub-centimeter precision. This proof-of-concept system therefore emphasizes demonstrating the feasibility of FTIR–ToF integration, leaving resolution optimization for targeted high-accuracy deployments.

Although the present work focuses on a single-sensor configuration (Figure 2), the same methodology can be scaled to larger or more complex sensing areas through alternative placement strategies, which we define below:

- Single-sensor configuration (current setup): The ToF unit is positioned at one edge of the light guide, providing a compact and cost-effective solution for localized touch detection. This arrangement minimizes hardware complexity but limits the sensing area to the field of view of a single device.
- Dual-opposite sensor placement: Positioning two ToF sensors at opposite edges of the light guide can approximately double the measurable length, provide redundant coverage, and partially mitigate occlusion effects caused by touch objects blocking the optical path.
- Multi-sensor edge array: For large-area, multi-touch applications, multiple sensors can be placed along the perimeter. This increases spatial resolution and coverage uniformity, while enabling simultaneous detection of multiple touch points without significant blind spots.
- Reflective edge termination: Applying reflective coatings or mirrored terminations at selected edges can extend the effective sensing range without adding additional sensors. This approach redirects light within the light guide to otherwise unreachable regions, though it may introduce a trade-off in signal intensity and an increase in internal reflections.

The choice among these configurations depends on application-specific requirements for resolution, cost, available form factor, and environmental robustness. While optimization of these layouts lies beyond the scope of the present study, the underlying design principles follow directly from the single-sensor results presented here.

It should be noted that the multi-touch capability of the proposed system is not fundamentally constrained by the number of sensing zones in the ToF hardware. Instead, performance is determined by factors such as occlusion effects, spatial resolution, and the optical quality of the light guide (including refractive index stability, surface finish, and internal homogeneity). With appropriate material selection and system calibration, the architecture can, in principle, support an arbitrary number of simultaneous touch points within the instrumented area.

#### 3.3.3. Economic Aspects

A comparative analysis of FTIR-based multi-touch sensor technologies highlights the cost-effectiveness of the novel FTIR–ToF integration presented in this work. Table 1 provides a price-per-square-inch comparison of several representative interactive display technologies, along with their reported display and touch resolution where available. The data illustrate the significant variation in cost depending on the sensing approach and display size. For example, traditional FTIR systems such as Perceptive Pixel by Microsoft range from $8.81 to $19.58 per square inch, depending on screen size. Similarly, systems like the SMART Table and Touch Revolution’s Fusion Touch Displays offer lower costs of $3.36 and $2.10 per square inch, respectively, reflecting simpler hardware configurations and reduced component costs.

In contrast, the FTIR–ToF prototype developed in this study achieved a markedly lower cost per area of approximately $0.74 per square inch based on the current single-sensor implementation. This high cost-effectiveness results from the integration of a compact ToF sensor with a PMMA-based light guide, eliminating the need for multiple infrared emitters and cameras typical of traditional FTIR designs. In addition to reducing the bill of materials, this architecture lowers power consumption and simplifies assembly.

While the absolute prices in Table 1 reflect historical values reported in manufacturer specifications or prior literature, they provide a consistent basis for comparing relative economic positioning. The modular nature of the ToF sensor integration and the adaptability of PMMA light guides enable straightforward scaling to larger formats, potentially maintaining low unit-area costs even for large multi-touch surfaces. Furthermore, the inclusion of ToF-based depth data improves robustness under challenging ambient lighting conditions, which can reduce calibration and maintenance overhead over the system’s lifetime.

Overall, the proposed FTIR–ToF architecture offers a competitive and scalable alternative to existing FTIR-based multi-touch displays, particularly for applications where moderate spatial resolution, environmental robustness, and cost efficiency are prioritized over sub-millimeter precision.

## 4. Conclusions

This work has demonstrated the integration of Frustrated Total Internal Reflection (FTIR) technology with a compact time-of-flight (ToF) sensor to realize a scalable, robust, and cost-efficient multi-touch interface. The proposed setup, using a 2 mm-thick PMMA light guide with high transmittance at the 940 nm operating wavelength, enables direct coupling to the ToF sensor and achieves a substantial field of view alongside linear resolution of approximately 10 mm and angular resolution up to $3.5^{{}^{\circ}}$. These characteristics confirm the suitability of the approach for medium- to large-scale interactive surfaces, including those requiring multi-point touch detection.

Performance validation experiments addressed key physical and optical constraints. Signal decay measurements along the optical axis revealed an exponential attenuation pattern with a decay constant of $k=0.0245\;{\mathrm{mm}}^{-1}$, defining an effective sensing range of approximately 200 mm for the present configuration. Axial occlusion tests showed up to 70% suppression of a rear object’s signal in stacked-touch conditions, underscoring the role of optical path obstruction in limiting multi-touch performance. Comparative tests between flat and curved light guides indicated negligible variation in peak response, suggesting geometric robustness of the sensing principle. Ambient infrared interference tests confirmed susceptibility to external 940 nm sources, with up to 66% amplitude reduction under strong illumination—highlighting the need for optical shielding or adaptive compensation in uncontrolled environments.

The system’s modular architecture naturally supports alternative sensor placement strategies, such as dual-opposite or multi-edge arrays, which could extend sensing range, improve spatial coverage, and mitigate occlusion effects. Furthermore, multi-touch capability is not fundamentally limited by the sensing method but rather by application-specific constraints such as light guide quality, spatial resolution, and environmental robustness.

An important complement to system-level optimization is material selection. As demonstrated by Aulika et al. [9], systematic baseline characterization of candidate light guide materials can provide early-stage evaluation of refractive index, absorption, and scattering properties. Integrating such a process into FTIR–ToF system development would enable informed material choices that maximize detection range and stability.

Economically, the presented proof of concept achieves a price-per-square-inch of approximately $0.74—an order of magnitude lower than many commercial FTIR-based systems—while maintaining functional multi-touch capability. Combined with the reduced component count, lower power consumption, and compact form factor, this approach represents one of the most cost-effective FTIR-type sensing architectures to date.

In conclusion, the FTIR–ToF integration presented here establishes a promising foundation for next-generation interactive displays, public kiosks, control panels, and industrial human–machine interfaces. Future work should focus on enhancing IR filtering, optimizing optical coupling, refining reconstruction algorithms, and exploring larger-area multi-sensor configurations to fully exploit the scalability potential demonstrated in this study.

## Figures and Tables

**Table 1:** Price-per-square-inch analysis for FTIR-based interactive displays, now including reported display/touch resolution. “n/s” = not specified in the cited source. Prices reflect values reported in cited sources at the time of publication and may not represent current market conditions.

Product Name	Diagonal (in)	Price ($)	Area (sq. in.)	Price/sq. in. ($)	Resolution	Reference
Perceptive Pixel by Microsoft (55”)	55	80,000	4085.20	19.58	Display: 1920 × 1080 Touch: sub-mm (claimed)	[14,15]
Perceptive Pixel by Microsoft (82”)	82	80,000	9080.61	8.81	Display: 1920 × 1080 Touch: sub-mm (claimed)	[14,15]
SMART Table by SMART Technologies	42	8000	2382.24	3.36	Display: 1920 × 1080 Touch: n/s	[16]
Touch Revolution’s Fusion Touch Displays	42	5000	2382.24	2.10	Display: 1024 × 600 Touch: ∼1 mm (center linearity)	[17]
3D Touch Surface (FTIR prototype)	n/a	100	n/a	n/a	Display: n/s Touch: 10 mm	[18]
This technology (FTIR–ToF, single sensor)	12	45	60.45	0.74	Display: – Touch: ∼10 mm; up to 3.5°	–

## Data Availability

The original contributions presented in this study are included in the article/Supplementary Material. Further inquiries can be directed to the corresponding author.

## Supplementary Materials

The following supporting information can be downloaded at https://www.mdpi.com/article/10.3390/s25175503/s1. Video S1: Real-time demonstration of flat prototype multi-touch detection (Flat_PMMA.mp4). Video S2: Real-time demonstration of curved prototype multi-touch detection (Curved_PMMA.mp4). Script S1: Python implementation of histogram-based FTIR–ToF signal reconstruction (tmf8828_reconstruction_demo.py).
